# Relapse of severe acute malnutrition among children discharged from outpatient therapeutic program in western Ethiopia

**DOI:** 10.1186/s12887-023-04269-7

**Published:** 2023-09-02

**Authors:** Endalkachew Befekadu Teshale, Yakob Desalegn Nigatu, Tefera Darge Delbiso

**Affiliations:** 1 Gambella Region Health Office, Gambella, Ethiopia; 2 Department of Public Health Nutrition and Dietetics, School of Public Health, Addis Ababa University, Addis Ababa, Ethiopia

**Keywords:** Severe acute malnutrition, Relapse, Discharge, Therapeutic program

## Abstract

**Background:**

Children with severe acute malnutrition (SAM) without complication are treated in the outpatient therapeutic program (OTP) and the program has been reported to be effective. However, relapse post-discharge from the program is poorly defined, and scarcely evaluated across programs and research. The objective of this study is to assess the prevalence of SAM among children post-discharge from the OTP and to identify factors associated with SAM relapse in Gambella Region, Western Ethiopia.

**Methods:**

We conducted a facility-based cross-sectional study among 208 children aged 6–59 months who have been discharged from the OTP as cured. Baseline data were collected from caregivers using structured questionnaire. Child anthropometry and oedema was measured. The association between SAM relapse and the risk factors were assessed using bivariate and multivariable logistic regression models.

**Results:**

The prevalence of SAM relapse was 10.1% (95% CI: 5.8–14.0%). The odds of SAM relapse was significantly higher in children with mothers who had no exposure to education and promotion about infant and young child feeding (IYCF) practices (OR = 5.7; 95% CI: 1.3–12.6), children who were not fully immunized for their age (OR = 8.0; 95% CI: 3.8–23.4), and children with mid-upper arm circumference (MUAC) at discharge of < 12.5 cm (OR = 4.4; 95% CI: 2.1–12.8) than their counterparts.

**Conclusions:**

To reduce SAM relapse, the OTP programs should avoid premature discharge and consider provision of supplementary food for children with low MUAC at discharge. Further, the OTP discharge criteria should consider both the anthropometric indicators – weight-for-height/length z-score (WHZ) and MUAC – and the absence of bilateral pitting oedema irrespective of the anthropometric indicator that is used during admission. Promotion of nutrition education and improving child immunization services and coverage would help reduce SAM relapse.

**Supplementary Information:**

The online version contains supplementary material available at 10.1186/s12887-023-04269-7.

## Background

Severe acute malnutrition (SAM), which is manifested in the form of severe wasting or oedema, is a serious public health problem in under-5 children. Globally, in 2020, 13.6 million children under-5 years of age were severely wasted [1]. SAM is most prevalent in developing countries, including Ethiopia [1–3]. According to a recent national survey, the prevalence of SAM in Ethiopia stagnated at 11% in 2023 [2] from the 10% mark in 2016 [4]. According to the World Health Organization (WHO), SAM in children aged (6–59 months) is diagnosed with weight-for-height/length z-score (WHZ) <-3, mid-upper arm circumference (MUAC) < 11.5 cm or presence of bilateral pitting oedema [5, 6]. Children with SAM are about nine times more at risk of mortality than non-malnourished children [7], and survivors suffer impairment in physical growth, motor development, and are at high risk to communicable and non-communicable diseases [1, 3, 8–10].

Community-based management of acute malnutrition (CMAM) is widely used to treat children with SAM. This approach has both a community-based outpatient therapeutic feeding program and a facility-based inpatient care. The decision of where to manage SAM cases depends on the presence of complication or not [6, 10]. Children with uncomplicated SAM who passed the appetite test and free from medical complications are treated in the outpatient therapeutic program (OTP) by providing the necessary medications and nutritional supplements, such as antibiotics and ready-to-use therapeutic foods (RUTF), and health and nutrition education [7]. Details of treatments provided is presented in [Additional file 1]. According to the national guideline for the management of acute malnutrition in Ethiopia [11], children are discharged as recovered from OTP for SAM treatment when they reach a MUAC ≥ 12.5 cm or WHZ ≥ -2, clinically well and alert, and have no bilateral pitting oedema for two consecutive visits. Children who fail the appetite test and have medical complications are referred to the inpatient program because these are signs of severe metabolic malnutrition and thus the children are at immediate risk of death [11].

The CMAM programs are effective as it achieves the internationally agreed standards for recovery rate of > 75%, death rate of < 10%, default rate of < 15%, length of stay of < 4 weeks and coverage of > 50–70% in sub-Saharan Africa, including Ethiopia [12–14]. However, the CMAM program achievements are short-range, which focuses on achieving immediate recovery while the children are in the program and does not indicate sustainability of recovery post discharge. Furthermore, some studies documented that those children who were discharged from OTP as cured are susceptible for morbidity and mortality afterwards [15, 16]. Monitoring of children periodically post-discharge from SAM treatment programs to avoid relapse (i.e., cured within the past 3 months and now meets the admission criteria for OTP) is strongly recommended [6, 9]. With these backgrounds, the aim of this study is to estimate the prevalence of SAM among children post discharge from the OTP and its associated factors in Gambella region of Ethiopia, one of the regions with the highest prevalence of acute malnutrition (14.1%) according to the Ethiopian Demographic and Health Survey (EDHS) [4].

## Methods

### Study setting

The study was conducted in Gambella region (Gambella town and the nearby districts), which is located in the western tip of Ethiopia bordering South Sudan in the West, South, and North. Gambella is one of the largest refugee-hosting (mainly South Sudanese refugees) and conflict-ridden (mainly ethnic violence and insecurity) regions in Ethiopia. The study area has one public referral hospital, one primary hospital, three public health centers, 13 health posts and 11 private clinics. In the study area, health posts do not treat SAM cases; rather they refer to either the health centers or the primary hospitals. Accordingly, three public health facilities – Gambella town primary hospital, Abol health center and Bonga health center – provide routine OTP service in the study area.

### Study design and period

We conducted a facility-based cross-sectional study from April to June 2020 among children (6–59 months) of age who have been discharged as cured from OTP of the selected three health facilities between January 2019 and January 2020.

### Eligibility criteria

We included children (6–59 months) of age who have been discharged as cured from the selected OTP with mothers or caregivers pair. We excluded children with physical deformity or congenital malformation which alters accurate anthropometric measurement.

### Sample size calculation and sampling procedures

We calculated the sample size using a single population proportion formula [17] assuming a SAM relapse (WHZ<-3) prevalence of 18.1% from the previous study conducted in Nigeria [18], a 95% level of confidence, and a 5% margin of error. The calculated sample size becomes 227 and by considering a non-response rate of 10% the final sample size becomes 250 children.

The study was conducted in all the three public health facilities, which provide routine OTP service in Gambella town and nearby districts. To recruit children for the study, the calculated sample size was proportionately allocated to the three health facilities based on the previous one-year data of discharged children. Accordingly, 113 children from Gambella town primary hospital, 75 from Bonga health center, and 62 from Abol health center were included in the study. Then, using children’s OTP registration as a sampling frame, children were selected randomly using lottery methods.

### Data Collection tools and measurements

We started the data collection by preparing a checklist using the OTP multi-chart and registration logbook which has been used in the Ethiopia [11]. From the registration logbook, important variables, such as sex, age, weight, height, MUAC, date of admission, date of discharge, and address were captured. Then, we identified children discharged as cured using the address. We interviewed the mothers or caregivers about the socio-demographic, nutritional and clinical information using a structured questionnaire adopted from the EDHS [4] and similar studies. Following the interview, the mothers or caregivers brought the children to health facilities for anthropometric measurements. The age of children was determined using mothers or caregivers recall of the date of birth, birth certificates, and/or immunization cards.

Child feeding practices were assessed using standard indicators for assessing infant and young child feeding (IYCF) practices tool [19]. The tool includes questions related to initiation of breastfeeding, when the child received colostrum, exclusive breastfeeding, complementary feeding, and continuity of breastfeeding (for children ≤ 2 years of age). Child health status in the previous one month before the survey date was captured from the mothers or caregivers: for children who were ill, symptom of the illness and status of hospitalization was recorded. HIV status of the child was captured from the test result recorded in the OTP registration.

We measured height for children ≥ 2 years of age and length for children < 2 years of age using a portable measuring board to the nearest 0.1 cm. We weighted children ≥ 2 years of age alone; and the younger children together with their caregivers and subtracted the child’s weight. We used a digital scale to the nearest 0.01 kg. Anthropometric measurements were taken twice and the difference between replicate results was checked. For values above the allowable difference (i.e., 0.5 kg for weight and 1.0 cm for height [11]), the measurements were repeated and then the average of the two measurements was taken. Oedema was checked by applying thumb pressure on top of both feet for 3 seconds to see if it leaves a pit (indentation) in the foot after the thumb is lifted [15].

The data collection was undertaken by six data collectors (3 urban health extension nurses and 3 diploma nurses) and two supervisors (health officers) who were working in OTP centers and had previous experience in similar assignments. Two days intensive training was given to data collectors and supervisors. The questionnaire was pretested on 5% of the total sample at Itang health center, which is located 38 km from Gambella town and nearby districts and it is not part of the study. Ambiguous questions were revised accordingly. The supervisors and the principal investigator monitored the data collection process and the collected data on a daily basis.

### Study variables

The outcome variable of the study is SAM relapse among children who have been discharged from OTP 3–12 months prior to the survey date. It is dichotomized into two: relapsed in to SAM (if WHZ <-3) and/or presence of bilateral pitting oedema, and not relapsed otherwise [6, 9].

Explanatory variables include socio-demographic variables, such as child characteristics (age and sex), mothers characteristics (age, marital status, level of education, and occupational status), household characteristics (family size, number of under-5 children in the household, and household income – defined according to the World Bank atlas method [20] where households with an average monthly income of ≤ 2424 Ethiopian Birr (ETB) are categorized as low income, 2425–9475 ETB low-middle income, 9476–29,362 ETB upper-middle income, and ≥ 29,363 ETB high income), nutrition and food security-related variables (WHZ and MUAC on admission and discharge, household food insecurity – measured using Household Food Insecurity Access Scale [21]), ever-exposure to education and promotion about IYCF practices, and clinical variables of the child (full immunization status according to [22], illness history one month prior to the survey, symptoms of the illness, hospitalization status one month prior to the survey, and HIV status).

### Data management and statistical analysis

Anthropometric indices were derived based on the WHO 2009 child growth standard [5] using WHO Anthro software 3.2.2. Prior to analysis, inconsistencies, missing values, outliers and values outside of the range were screened and cleaned. Data were summarized and presented in frequency tables. For categorical explanatory variables, their association with SAM relapse was assessed using Chi-square and Fisher exact test.

To determine the association between SAM relapse and the explanatory variables, we used bivariate and multivariate logistic regression analysis. First, a bivariate analysis was conducted between the outcome variable and the explanatory variables. Then explanatory variables found to be significant at 5% are included in the multivariate logistic regression analysis. Collinearity between the explanatory variables were evaluated using variance inflation factor (VIF) and found to be non-substantial (VIF < 3); the model goodness-of-fit was evaluated using Hosmer and Lemeshow test and found to be a good-fit (p-value = 0.79). Finally, the Odds Ratios (OR) and their 95% confidence intervals (CI) are reported for each of the variable in the final model. Variables with a p-value of < 0.05 in the final model were declared statistically significant.

### Ethics approval and consent to participate

This study was conducted according to the guidelines of the Helsinki Declaration. All procedures involving research study participants were approved by the research and ethical review board of the School of Public Health, Collage of Health Sciences, Addis Ababa University (Ref. No. 599; SPH/20/12). Verbal informed consent was obtained from the mothers and parental/guardian assent was obtained for the children. A child who found to have SAM and who is not fully vaccinated for his/her age was linked to health facilities. Nutritional education and promotion on the IYCF practices was given to all mothers or caregivers who participated in the study.

## Results

### Characteristics of the study participants

Two hundred fifty children who have been discharged from OTP as cured were traced. Of which, we able to collect data from 208 children-mother pair: 7 (2.8%) died and 35 (14.0%) gave either untraceable address or consent withdrawn making the response rate 83.2%. The median age of the children was 24.4 months (Interquartile Range (IQR): 20.5–29.9) – 88% were in the age group 13–36 months; and about half were male. The median age of the mothers was 27 (IQR: 21–31); 80% were married; 70% were literate; and 12.5% were working during the survey period. About 89% of the households fall under the low-middle income category; and 46% were moderately to severely food insecure. In the bivariate analysis, under-5 children in the household and food security status were significantly associated with SAM relapse and thus included in the multivariable logistic regression (Table 1).

**Table 1:** Characteristics of children (6–59 months) and their mothers or caregivers post discharge from OTP in Gambella town and nearby districts, Ethiopia, April – June 2020

Characteristics	Children or Mother, No. (%)			
	All (N = 208)	SAM relapsed (n = 21)	SAM not relapsed (n = 187)	P-values
Child sex				
Female	102 (49.0)	12 (57.1)	90 (48.1)	0.433
Male	106 (51.0)	9 (42.9)	97 (51.9)	
Child age during admission (in months) [Median (IQR)]	24.4 (20.5– 29.9)	27.2 (19.4– 33.8)	24.4 (20.5– 29.5)	
6–12	2 (1.0)	0 (0.0)	2 (1.1)	0.393
13–24	93 (44.7)	7 (33.3)	86 (46.6)	
25–36	90 (43.3)	10 (47.6)	80 (42.8)	
37–48	15 (7.2)	2 (9.5)	13 (7.0)	
49–59	8 (3.8)	2 (9.5)	6 (3.2)	
Maternal age (in years) [Median (IQR)]	27 (21–31)	25 (19–33)	27 (21–31)	
15–24	89 (42.8)	10 (47.6)	79 (42.2)	0.422
25–34	101 (48.6)	8 (38.1)	93 (49.7)	
> 35	18 (8.7)	3 (14.3)	15 (8.0)	
Maternal educational level				
No education	99 (47.6)	13 (66.7)	86 (46.0)	0.466
Primary	65 (31.3)	6 (28.6)	59 (31.6)	
Secondary and above	39 (21.1)	2 (9.5)	42 (22.5)	
Marital status of the mother				
Currently married	167 (80.3)	16 (76.2)	151 (80.7)	0.573
Currently single	41 (19.7)	5 (23.8)	36 (19.3)	
Working status of the mother				
Working	26 (12.5)	5 (23.8)	21 (11.2)	0.406
Housewife	117 (56.2)	10 (47.7)	107 (57.2)	
Unemployed	65 (31.3)	6 (28.6)	59 (31.5)	
Family size				
<=5	63 (30.3)	4 (19.0)	59 (31.6)	0.237
> 5	145 (69.7)	17 (81.0)	128 (68.4)	
Under-5 children in the household				
<=2	157 (75.5)	7 (33.3)	150 (80.2)	< 0.001
> 2	51 (24.5)	14 (66.7)	37 (19.8)	
Household income category				
Low-income (≤ 2424 ETB)	8 (3.8)	1 (4.7)	7 (3.7)	0.858
Low-middle (2425–9475 ETB)	185 (88.9)	19 (90.5)	166 (88.8)	
Upper-middle (9476–29,362 ETB)	15 (7.2)	1 (4.7)	14 (7.5)	
Household food security status				
Food secured	14 (6.7)	1 (7.1)	13 (7.0)	0.021
Mildly insecure	98 (47.1)	4 (4.1)	94 (50.3)	
Moderately insecure	57 (27.4)	9 (15.8)	48 (25.7)	
Severely insecure	39 (18.8)	7 (17.9)	32 (17.1)	

p-values are based on Chi-square or Fishers exact test

### The prevalence of SAM relapses

The prevalence of SAM relapse was determined based on the time between discharge from OTP and survey date. Of the 208 children successfully traced, 21(10.1%) relapsed into SAM.

### Nutrition and clinical related characteristics of children post discharge from OTP

Table 2 shows nutritional and clinical related characteristics of children (6–59 months) post-discharge from OTP. All the mothers reported having breastfed their children: about 95% of the children put on breastfeeding within 24 h of birth (69.2% within the first hour); 60.1% exclusively breastfed in the first six months; and 91.3% fed colostrum within the first three days after birth. About 71% of children started complementary feeding timely (semisolid foods at 6 months of age); among ≤ 2 years old children, 28.4% were already stopped breastfeeding during the survey time; and 56.3% of the mothers or caregivers have exposure to education and promotion about IYCF practices. Regarding child’s immunization status, 88.5% were vaccinated – 72.6% were fully vaccinated for their age. The post-discharge illness history one month prior to the survey date shows, 64.4% of the children were ill mainly with symptoms of diarrhea and fever; and 15.7% were hospitalized for the illnesses. Among HIV test result reported children, 8.5% were HIV positive (Table 2).

Exclusive breastfeeding, colostrum feeding, caregiver’s exposure to education and promotion about IYCF practices, full immunization, illness history, and HIV status were independently associated with SAM relapse in the bivariate analysis and thus included in the multivalve logistic regression (Table 2).

**Table 2:** Nutrition and clinical related characteristics of children (6–59 months) post discharge from OTP (n = 208)

Characteristics	Children, No. (%)			
	All (N = 208)	SAM relapsed (n = 21)	SAM not relapsed (n = 187)	P-value
Initiation of breastfeeding				
Immediately (within 1 h of birth)	144 (69.2)	14 (66.7)	130 (69.5)	0.602
< 24 h	53 (25.5)	5 (23.8)	48 (25.7)	
> 24 h	11 (5.3)	2 (9.5)	9 (4.8)	
Exclusive breastfeeding				
Yes	125 (60.1)	8 (38.1)	117 (62.6)	0.030
No	83 (39.9)	13 (61.9)	70 (37.4)	
Colostrum				
Yes	190 (91.3)	14 (66.7)	176 (94.1)	0.001
No	18 (8.7)	7 (33.3)	11 (5.9)	
Timely initiation of complementary feeding				
Yes	148 (71.2)	16 (76.2)	132 (70.6)	0.591
No	60 (28.8)	5 (23.8)	55 (29.4)	
Continuity of breastfeeding (for ≤ 2 years) (n = 96)				
Continued breastfeeding	69 (71.6)	3 (37.5)	66 (75.0)	0.065
Stopped breastfeeding	27 (28.4)	5 (62.5)	22 (25.0)	
Ever-exposure to education and promotion about IYCF practices				
Yes	117 (56.2)	4 (19.0)	113 (60.4)	<0.001
No	91 (43.8)	17 (81.0)	74 (39.6	
Fully immunized (n = 172)				
Yes	151 (72.6)	7 (58.3)	144 (90.0)	0.008
No	21 (10.1)	5 (41.7)	16 (10.0)	
Illness history (one month prior to the survey)				
Yes	134 (64.4)	18 (85.7)	116 (62.0)	0.032
No	74 (35.6)	3 (14.3)	71 (38.0)	
Hospitalization status (one month prior to the survey) (n = 134)				
Yes	21 (15.7)	3 (16.7)	18 (15.5)	0.060
No	113 (84.3)	15 (83.3)	98 (84.5)	
HIV status (n = 130)				
Positive	11 (8.5)	4 (28.6)	7 (6.0)	0.031
Negative	119 (91.5)	10 (71.4)	109 (94.0)	

p-values are based on Chi-square or Fishers exact test

### Anthropometric indicators during admission and discharge

The majority of the children (82.7%) were admitted with WHZ<-3 and 63.9% with MUAC < 11.5 cm. The nutritional status of the children significantly improved during discharge. During discharge, 68.8% of the children had WHZ ≥-2 and 63.0% had MUAC ≥-12.5 cm. MUAC on discharge was significantly associated with SAM relapse and thus included in the multivariable logistic regression. Of the relapsed children, 14(66.7%) were discharged less than 6 months prior to the survey date (Table 3). No oedema case was recorded on admission and during the survey date.

**Table 3:** Antropometric indicators of children (6–59 months) on admission and on discharge from OTP

Nutritional status indicators	Children, No. (%)			
	All (N = 208)	SAM relapsed (n = 21)	SAM not relapsed (n = 187)	P-value
WHZ on admission				
<-3	172 (82.7)	17 (81.0)	155 (82.9)	0.770
≥-3	36 (17.3)	4 (19.0)	32 (17.1)	
MUAC on admission				
< 11.0 cm	60 (28.8)	7 (33.3)	53 (28.3)	0.691
11.0–11.49 cm	73 (35.1)	9 (42.9)	64 (34.2)	
11.5–12.5 cm	66 (31.7)	5 (23.8)	61 (32.6)	
> 12.5 cm	9 (4.3)	0 (0)	9 (4.8)	
WHZ on discharge				
<-2 to >-3	65 (31.3)	8 (38.1)	57 (30.5)	0.770
≥-2	143 (68.8)	13 (61.9)	130 (69.5)	
MUAC on discharge				
< 12.5 cm	77 (37.0)	14 (66.7)	63 (33.7)	0.003
≥-12.5 cm	131 (63.0)	7 (33.3)	124 (66.3)	
Time between discharge from OTP and survey date				
3–<6 months	118 (56.7)	14 (66.7)	104 (55.6)	0.652
6 –<9 months	51 (24.5)	3 (14.3)	48 (25.7)	
9–12 months	39 (18.8)	4 (19.0)	35 (18.7)	

p-values are based on Chi-square or Fishers exact test

### Factors independently associated with SAM relapse

Statistically significant variables in the bivariate analysis (under-5 children in the family, food security status, exclusive breastfeeding, colostrum, exposure to education and promotion about IYCF practices, full immunization status, illness history, and HIV status) were included in the multivariable logistic regression. Despite the time between discharge from OTP and survey date was not significant at bivariate analysis, we included it in the multivariable logistic regression for its theoretical relevance. Three variables are significantly associated with SAM relapse in multivariable logistic regression – exposure to education and promotion about IYCF practices, immunization status, and MUAC at discharge. Accordingly, children of mothers who had no exposure to education and promotion about IYCF practices were 5.7 times more likely to relapse into SAM (OR = 5.7; 95% CI: 1.3–12.6); children who were not fully vaccinated for their age were 7.9 times more likely to relapse into SAM (OR = 7.9; 95% CI: 3.8–23.4); and children with MUAC at discharge < 12.5 cm were 4.4 times more likely to relapse into SAM (OR = 4.4; 95% CI: 2.1–12.8) (Table 4).

**Table 4:** Multivariable-adjusted binary logistic regression analysis of factors associated with SAM relapse

Characteristics	SAM relapsed (%)	OR (95% CI)	p-value
Ever-exposure to education and promotion about IYCF practices			
Yes	4 (19.0)	1.0	0.003
No	17 (81.0)	5.7 (1.3; 12.6)	
MUAC at discharge			
< 12.5 cm	14 (66.7)	4.4 (2.1; 12.8)	0.040
>=12.5 cm	7 (33.3)	1.0	
Fully immunized			
Yes	7 (58.3)	1.0	0.010
No	5 (41.7)	7.9 (3.8; 23.4)	
Time between discharge from OTP and survey date			
3–<6 months	14 (66.7)	1.0	0.453
6 –<9 months	3 (14.3)	2.1 (0.59; 7.85)	
9–12 months	4 (19.0)	1.2 (0.36; 3.82)	

Adjusted for number of under-5 children, food security status, exclusive breastfeeding, colostrum feeding, illness history, and HIV status

## Discussion

Our study aimed at assessing the prevalence of SAM relapse and its associated factors among children (6–59 months) of age who have been discharged from OTP as cured in Gambella town and nearby districts of Ethiopia. Children were assessed after 3–12 months of discharge from OTP. The study showed a SAM relapse prevalence of 10.1% (95% CI: 5.8–14.0%); 6.7% of the relapse occurred among children discharged < 6 months prior to the survey date.

The SAM relapse prevalence of our study is in accordance with similar studies from the Democratic Republic of Congo [23] and Burkina Faso [24], which reported 11.1% and 10.5% relapse prevalence at 6-month and 12-month follow-up periods, respectively. However, the relapse prevalence is below Nigeria’s 24% at 6-month follow-up [25], but above Malawi’s 1.9% at 3-month follow-up [26]. Various interventions have been implemented to reduce SAM relapse, which may explain the observed difference. Particularly, supplementary feeding following discharge help ensure children reach a state of adequate nutrition. For example, in Malawi, children provided with a two-week supply of program ration at discharge contributed to the reported low SAM prevalence [26]. An evaluation study conducted in Ethiopia also confirmed the effectiveness of supplementary feeding in increasing the WHZ [27]. A cluster randomized control trial conducted in the Democratic Republic of Congo found cash transfer to be effective in preventing relapse [23]. However, the Ethiopia CMAM program do not have a supplementary feeding option available for children discharged from the centers. Furthermore, the current strategy in Ethiopia is to provide supplementary feeding to selected chronically food insecure districts, overlooking moderate food insecure districts where households with chronic food insecurity might exist [4]. In our study, for example, only 6.7% of the households were food secured.

Children with mothers who had no exposure to any education and promotion about IYCF practices were 5.7 times more likely to have SAM relapse. This finding is supported with studies conducted in Ethiopia and India where children with mothers who had no exposure to nutritional counselling about IYCF practice are found more likely to relapse [28, 29]. Knowledge about nutrition and feeding practices are more important than formal maternal education to affect the nutritional status of the child [30]. Therefore, nutritional education and promotion that focuses on improving IYCF practices is recommended due to its effectiveness in treating children with acute malnutrition [31]. Yet, it is noted that only 56.3% of the mothers or caregivers had exposure to education and promotion about IYCF practices in our study.

Full Immunization was associated with SAM relapse; children who were not fully immunized for their age were 7.9 times more likely to have SAM relapse. Studies conducted in Burkina Faso [24] and India [30] corroborate our findings. Vaccines support the development of the immune system and reinforces immunity that prevents recent infections [32], thus children who are fully vaccinated are less likely to be affected by health problems that lead to SAM relapse. Improving child immunization services and coverage would help reduce the prevalence of relapse.

Children with lower MUAC at discharge (< 12.5 cm) were 4.4 times more likely to have SAM relapse. This finding is in agreement with studies done in Burkina Faso [24] and Malawi [26] as children with lower MUAC at discharge were found more likely to have SAM relapse. Children with lower MUAC at discharge may have comorbidities and underlying biological deficit that take longer to recover immunologically and physiologically. These problems may not be captured using anthropometric measurement. As a result, the child may remain susceptible to infections and thereby relapse [8, 15]. We thus recommend the OTP discharge criteria to consider both the anthropometric indicators – WHZ and MUAC – and the absence of bilateral pitting oedema, against the existing Guideline for the Management of Acute Malnutrition in Ethiopia [11] where the anthropometric indicator that is used to identify and confirm SAM on admission also used to assess whether a child has reached nutritional recovery.

Our study has some limitations. First, the cross-sectional nature of the study did not allow us to establish cause-effect relationship between SAM relapse and its predictors. Second, the information gathered from the mothers retrospectively will likely to have a recall bias. However, we have included probing questions to obtain reliable information. Third, we cannot be sure how the 14.0% of children untraced might have affected the result. Fourth, caution must be taken when extrapolating our study findings to children discharged from OTP in other regions due to contextual differences. Finally, we recommend further studies using longitudinal data or systematic reviews to establish the cause of SAM relapse considering the aforementioned limitations.

## Conclusions

We found that the prevalence of SAM relapse in children discharged from OTP as cured in Gambella town and nearby districts of Ethiopia was 10.1% (95% CI: 5.8–14.0%). Being children of mothers who had no exposure to education and promotion about IYCF practices, children who are not fully vaccinated for their age, and children with MUAC at discharge < 12.5 cm had higher odds of SAM relapse. We recommend programs to avoid premature discharge (i.e., discharge of children with MUAC < 12.5 cm) and to consider provision of supplementary food for children with low MUAC at discharge.

### Electronic supplementary material

Below is the link to the electronic supplementary material.

Supplementary Material 1

## Data Availability

All data generated or analyzed during this study are included in this manuscript.

## List of abbreviations

CI: Confidence Interval
CMAM: Community-based Management of Acute Malnutrition
EDHS: Ethiopian Demographic and Health Survey
IQR: Interquartile Range
IYCF: Infant and Young Child Feeding
MUAC: Mid-upper Arm Circumference
SAM: Severe Acute Malnutrition
OTP: Outpatient Therapeutic Program
OR: Odds Ratios
RUTF: Ready to Use Therapeutic Foods
VIF: Variance Inflation Factor
WHO: World Health Organization
WHZ: Weight-for-Height/length z-score
