# Supplementary material for: Relapse of severe acute malnutrition among children discharged from outpatient therapeutic program in western Ethiopia
Source: BMC Pediatr. 2023 Sep 2;23:441. doi: 10.1186/s12887-023-04269-7 (PMC10474695; doi:10.1186/s12887-023-04269-7)
Supplement: Supplementary file 1 — Supplementary Material 1 [file 12887_2023_4269_MOESM1_ESM.docx]

Additional file 1:

Table A: Summary of medications received

| Name of Medication | When to Give | Age | Dose | Prescription |
| --- | --- | --- | --- | --- |
| AMOXICILLIN | On admission of all SAM patients | All ages | 25 mg/kg, every 12 hours, for 5 days. | Oral: 25 mg/kg, every 12 hours, for 5 days |
| ANTIMALARIAL | On admission if positive malaria test | All ages | Refer to the National Malaria Guidelines | Refer to the National Malaria Guidelines |
| ALBENDAZOLE or MEBENDAZOLE | After 1 week for treatment in OTP | 24- 59 months | Albendazole 400 mg Mebendazole 500 mg | Single dose, orally |
| MEASLES VACCINE | On the fourth week if the child has not yet received the measles vaccine | 9–59 months | Refer to the National Expanded Programme on Immunisation (EPI) Guidelines | Refer to the National EPI Guidelines |
| OTHER VACCINES | Update vaccinations based on EPI schedule | All ages | Refer to the National EPI Guidelines | Refer to the National EPI Guidelines |

Table B: RUTF amount and frequency

Table below shows how to determine the amount of RUTF to give at each weekly visit, based on the patient’s weight.

Reference Table (92 g Sachet Containing 500 Kcal)

| Weight of Child (kg) | Sachets per Day | Sachets per Week |
| --- | --- | --- |
| 3.5 - 3.9 | 1 and half | 11 |
| 4.0 – 5.4 | 2 | 14 |
| 5.5 – 6.9 | 2 and half | 18 |
| 7.0 – 8.4 | 3 | 21 |
| 8.5 – 9.4 | 3 and half | 25 |
| 9.5 – 10.4 | 4 | 28 |
| 10.5 – 11.9 | 4 and half | 32 |
| ≥ 12 | 5 | 35 |
